# Comparison of autism spectrum disorder surveillance status based on two different diagnostic schemes: Findings from the Metropolitan Atlanta Developmental Disabilities Surveillance Program, 2012

**DOI:** 10.1371/journal.pone.0208079

**Published:** 2018-11-30

**Authors:** Lisa Wiggins, Deborah Christensen, Kim Van Naarden Braun, Lisa Martin, Jon Baio

**Affiliations:** National Center on Birth Defects and Developmental Disabilities, Centers for Disease Control and Prevention, Atlanta, GA, United States of America; Chinese Academy of Medical Sciences and Peking Union Medical College, CHINA

## Abstract

For the first time, the Autism and Developmental Disabilities Monitoring Network (ADDM) at the Centers for Disease Control and Prevention (CDC) reported prevalence estimates based on two different diagnostic schemes in the 2014 surveillance period. Results found substantial agreement between surveillance case status based on Diagnostic and Statistical Manual of Mental Disorders–Fourth Edition–Text Revision (DSM-IV-TR) criteria and DSM-5 criteria ASD (kappa = 0.85). No study has replicated this agreement in another independent sample of surveillance records. The objectives of this study were to (1) replicate agreement between surveillance status based on DSM-IV-TR criteria and DSM-5 criteria for ASD, (2) quantify the number of children who met surveillance status based on only DSM-IV-TR criteria and only DSM-5 criteria for ASD, and (3) evaluate differences in characteristics of these latter two groups of children. The study sample was 8-year-old children who had health and education records reviewed for ASD surveillance in metropolitan Atlanta, GA in the 2012 surveillance year. Results found substantial agreement between child’s surveillance status using DSM-IV-TR criteria and DSM-5 criteria for ASD (kappa = 0.80). There were no differences in child race/ethnicity, child sex, or intellectual disability between surveillance status defined by DSM-IV-TR criteria and that defined by DSM-5 criteria. Children who met surveillance status based on DSM-IV-TR criteria, but not DSM-5 criteria, were more likely to have developmental concerns and evaluations in the first three years. Children who met surveillance status based on DSM-5 criteria, but not DSM-IV-TR criteria, were more likely to have been receiving autism-related services or previously diagnosed with ASD. These results suggest that surveillance status of ASD based on DSM-5 criteria is largely comparable to that based on DSM-IV-TR criteria, and identifies children with similar demographic and intellectual characteristics.

## Introduction

Autism spectrum disorder (ASD) is a developmental disorder characterized by deficits in social-communication and interaction skills and the presence of restricted interests and repetitive behaviors [1]. The estimated prevalence of ASD reported by the Centers for Disease Control and Prevention (CDC)’s Autism and Developmental Disabilities Monitoring (ADDM) Network has increased substantially, from about 6.7 per 1,000 8-year-old children in 2000 to about 16.8 per 1,000 8-year-olds in 2014 [2–3]. CDC thus highlights ASD as an urgent public health concern and notes that continued monitoring of the prevalence of ASD is important to inform policy and research decisions, and understand how characteristics of people with ASD change over time.

Since the inception of ASD surveillance in 1996 by the Atlanta-based ADDM site, CDC has consistently applied a rigorous and reliable method to estimate the prevalence of ASD in the population [4]. This method employs a standardized coding scheme based on criteria outlined in the Diagnostic and Statistical Manual of Mental Disorders–Fourth Edition–Text Revision (DSM-IV-TR) which is applied to behavioral information collected from health and education records [5]. The methods used to determine ASD surveillance case status do not rely solely on an ASD diagnosis or special education classification of autism. The records-based ASD surveillance method developed by CDC has fairly high positive predictive value (0.79) when compared to a comprehensive clinical evaluation [6] and provides the best possible ASD prevalence estimate currently available without conducting complete resource intensive, population screening and diagnostic clinical case confirmation [7].

In DSM-IV-TR, ASD comprises subtypes including autistic disorder, Asperger disorder, Childhood Disintegrative Disorder (CDD), Rett syndrome, and Pervasive Developmental Disorder–Not Otherwise Specified (PDD-NOS). CDD and Rett syndrome are often excluded from ASD prevalence estimates because of the rare occurrence of these conditions and discovery of a genetic mutation that causes Rett syndrome independent of ASD [8]. Every two years since the 2000 surveillance year, the ADDM Network has reported prevalence estimates among 8-year-olds for ASD, including autistic disorder, Asperger disorder, and PDD-NOS under the parameters of the DSM-IV-TR diagnostic criteria [2, 3, 9–14].

In its publication of the DSM-5 in 2013, the American Psychiatric Association made considerable changes to ASD diagnostic criteria that influence CDC ASD surveillance methods [1]. In DSM-5, ASD no longer comprises distinct subtypes but represents one singular condition defined by severity levels, or the level of functional support required by the individual. Another change from DSM-IV-TR to DSM-5 is that DSM-5 specifies that individuals with ASD must meet all three social criteria (i.e., deficits in social-emotional reciprocity, deficits in nonverbal communicative behaviors, and deficits in developing, understanding, and maintaining relationships) and two of four behavioral criteria (i.e., repetitive speech or motor movements, insistence on sameness, restricted interests, or unusual response to sensory input). Moreover, DSM-5 states that those with a previous DSM-IV-TR diagnosis of autistic disorder, Asperger disorder, or PDD-NOS should be given a DSM-5 diagnosis of ASD.

For the first time, the ADDM Network reported prevalence estimates based on both DSM-IV-TR and DSM-5 criteria in the 2014 surveillance period [3]. Results found substantial agreement between surveillance status based on DSM-IV-TR and DSM-5 definitions of ASD (kappa = 0.85). These findings compliment other studies that suggest DSM-5 changes do not result in significant changes in ASD prevalence estimates and improve diagnostic specificity without a compromise in diagnostic sensitivity [15,16]. They contrast with studies that suggest DSM-5 changes may produce substantially lower prevalence estimates due to the exclusion of very young children and/or those without intellectual disability (ID) [17–21].

No study has replicated ADDM DSM-IV-TR versus DSM-5 surveillance estimates in other independent samples of surveillance records. It is important to replicate these findings so that future reports of DSM-5 prevalence estimates can be considered within the context of historically presented DSM-IV-TR prevalence estimates. The objectives of this study were to (1) replicate agreement between surveillance status based on DSM-IV-TR criteria and DSM-5 criteria for ASD in another sample of surveillance records, (2) quantify the number of children who met surveillance status based on only DSM-IV-TR criteria and only DSM-5 criteria for ASD, and (3) evaluate differences in characteristics of these latter two groups of children.

## Materials and method

The study sample was 8-year-old children who had health and education records reviewed for ASD surveillance in metropolitan Atlanta, GA in the 2012 surveillance year as a part of the Metropolitan Atlanta Developmental Disabilities Surveillance Program (MADDSP). MADDSP is an on-going records-based surveillance system that monitors the prevalence of selected developmental disabilities among 8-year-old children in five counties of metropolitan Atlanta, GA. MADDSP was initiated in 1991 for surveillance of cerebral palsy, hearing loss, intellectual disability, and vision impairment, with ASD added in 1996. MADDSP serves as the framework for the ADDM Network which has replicated the MADDSP methodology across multiple US communities. Each ADDM site, including MADDSP, functions as a public health authority under the Health Insurance Portability and Accountability Act of 1996 Privacy Rule and meets applicable local Institutional Review Board and privacy and confidentiality requirements.

### Procedures used for DSM-IV-TR ASD surveillance

A child was eligible for MADDSP ASD surveillance if he/she: (1) was born in 2004 (i.e., was 8 years old at any point during the 2012 surveillance year), (2) had a parent or legal guardian who resided in the five-county metropolitan Atlanta area, and (3) received service for a developmental condition as evidenced by a discharge diagnosis, billing code, reason for referral, or education eligibility noted in evaluation records. Surveillance staff reviewed health and education records of eligible children for social deficits that indicated the child may be at risk for ASD (e.g., limited interest in other children or reduced eye contact). All records that contained a social deficit were abstracted to collect accounts of developmental history, descriptions of ASD symptoms, results of developmental tests, and documentation of co-occurring conditions diagnosed by the community professional who evaluated the child. All abstracted information was combined into one composite record if multiple records were abstracted for the same child.

Clinicians with advanced degrees and specialized training and experience in ASD then applied a standardized coding scheme to the abstracted data. An algorithm based on DSM-IV-TR diagnoses of autistic disorder, Asperger disorder, or PDD-NOS was applied to the results of the coding scheme. In order to meet surveillance status of autistic disorder, the child had six or more ASD symptoms noted, with at least two social symptoms, one communication symptom, and one behavioral symptom; and evidence of developmental concern noted by three years of age. In order to meet surveillance status of other ASD, the child had to have two or more ASD symptoms noted, with at least one social symptom and one communication or behavioral symptom; and evidence of a behavior or diagnosis that discriminates children with ASD from children with other developmental delays or disorders (DD). Agreement on DSM-IV-TR ASD surveillance case status among clinicians was 85%. Detailed descriptions of ASD surveillance status based on DSM-IV-TR criteria are found in Table 1.

**Table 1:** Determining autism spectrum disorder (ASD) surveillance status based on the Diagnostic and Statistical Manual of Mental Disorder–Fourth Edition–Text Revision (DSM-IV-TR).

Autistic disorder	Child had 1) six or more DSM-IV-TR symptoms coded, with at least a) two social symptoms, b) one communication symptom, and c) one behavioral symptom, and 2) evidence of developmental concern by three years of age.
ASD	Child had 1) two or more DSM-IV-TR symptoms coded, with at least a) one social symptom and b) one communication or behavioral symptom, and 2) at least one behavior that distinguishes children with ASD from children with other developmental delays or disorders (including an ASD diagnosis).^a^
Suspected ASD: high certainty the child has ASD (Classified as non-ASD)	Child met criteria for autistic disorder but did not have evidence of developmental concern by three years of age, or child met the first or second criteria for ASD, above, but not both; and the clinician reviewer noted his or her certainty the child had ASD as 4 or 5 on a 5-point Likert scale.
Suspected ASD: low certainty the child has ASD (Classified as non-ASD)	Child met criteria for autistic disorder but did not have evidence of developmental concern by three years of age, or child met the first or second criteria for ASD, above, but not both; and the clinician reviewer noted his or her certainty the child had ASD as 1, 2, or 3 on a 5-point Likert scale.
Disqualified ASD (Classified as non-ASD)	Child met surveillance status for autistic disorder or ASD, but clinician reviewer disqualified child upon first primary review or after consensus discussion with another reviewer (e.g., symptoms better accounted for by another disorder).
Not applicable (Classified as non-ASD)	Child did not meet surveillance status for autistic disorder, ASD, or Suspected ASD, or child had a documented diagnosis of CDD or Rett.

^a^Behaviors that distinguish children with ASD from children with other developmental delays or disorders are considered “red flags” for ASD that would prompt further evaluation if reported by a parent or observed by a healthcare professional. These discriminators are necessary for the DSM-IV-TR case definition of ASD.

It is important to note that a previous ASD diagnosis was not sufficient evidence to confirm DSM-IV-TR surveillance case status. Instead, the child had to have two or more ASD symptoms noted in service records, with at least one social symptom and one communication or behavioral symptom, in addition to an ASD diagnosis or another “autism discriminator.” Other autism discriminators were: oblivious to children, oblivious to adults, little or no interest in others, rarely responds to a familiar social approach, lack of showing objects of interest, loss of previously acquired skills, language consist primarily of echolalia or jargon, repeats extensive dialogue, absent of impaired imaginative play, markedly restricted interests, unusual preoccupation, insists on sameness, nonfunctional routines, excessive focus on parts, unusual visual inspection, movement preoccupation, and sensory preoccupation.

The clinician who applied the surveillance coding scheme rated his or her certainty that the child had ASD given all available information in service records. Degree of certainty was rated on a 5-point scale with one representing the least amount of certainty and five representing the greatest amount of certainty. Children who met ASD surveillance status with a certainty rating of one could be disqualified as an ASD case based on clinical judgment (e.g., symptoms accounted for by another disorder). Children who met ASD surveillance case status with a certainty rating of one (if not already disqualified), two, or three were sent to a second clinician for an independent review and coding; the two clinicians who reviewed the common record then met for a consensus discussion and decided final ASD surveillance status.

Certainty ratings were also applied to records of children who did not meet ASD surveillance status. These ratings were helpful in constructing the sampling scheme outlined in Table 2. For the purposes of this paper, ratings of four and five were combined to signify “high certainty” and ratings of one, two, or three were combined to signify “low certainty” in order to remain consistent with procedures outlined in the above paragraph.

**Table 2:** Sampling strategy for autism spectrum disorder (ASD) surveillance status based on documented ASD diagnosis and Statistical Manual of Mental Disorders–Fourth Edition–Text Revision (DSM-IV-TR).

	Documented DSM-IV-TR ASD Diagnosis Present				Documented DSM-IV-TR ASD Diagnosis Absent				
DSM-IV-TR surveillance status^a^	Probability of DSM-5^b^	DSM-IV-TR sample	Minimum % DSM-IV-TR sampled	DSM-5 sample	Probability of DSM-5^b^	DSM-IV-TR sample	Minimum % DSM-IV-TR sampled	DSM-5 sample	Total DSM-5 sample
Autistic disorder	High	447	10	48	Moderate	159	100	159	**207**
ASD	Uncertain	99	100	99	Uncertain	66	100	66	**165**
Suspected ASD: high certainty	Low	19	100	19	Low	33	100	33	**52**
Suspected ASD: low certainty	Low	10	100	10	Low	415	10	45	**55**
Disqualified ASD	Uncertain	18	100	18	Low	159	10	23	**41**
Not applicable	Uncertain	1	100	1	Very low	116	10	15	**16**
TOTAL		594	—	195		948	—	341	**536**

^a^See Table 1 for detailed description of surveillance status defined by DSM-IV-TR criteria

^b^Probability of DSM-5 is probability the child will meet DSM-5 social-behavioral criteria independent of a previous ASD diagnosis given literature review and clinical judgment.

### Procedures used for DSM-5 ASD surveillance

A DSM-5 coding scheme for ASD surveillance was developed by an independent body of ADDM-affiliated experts and then adapted and refined by a CDC-led clinical workgroup. In brief, an independent body of experts gathered behavioral exemplars from the DSM-IV-TR surveillance manual and other sources and re-coded exemplars according to DSM-5 criteria (e.g., language delay is coded as a diagnostic symptom in DSM-IV-TR and an associated feature in DSM-5; unusual sensory response is coded as an associated feature in DSM-IV-TR and a diagnostic feature in DSM-5). The re-coding of each exemplar was discussed in detail and questions were posed to members of the DSM-5 neurodevelopmental disorders workgroup. The re-coding process was then reviewed and discussed by a CDC-led workgroup of ADDM clinician reviewers. Agreement on ASD surveillance case status among clinicians was 100%, 97%, and 90% for the first three sets of reliability exercises for DSM-5 record-review coding.

It is important to note that a previous diagnosis of ASD was incorporated into the DSM-5 surveillance definition, since DSM-5 specifically states that those with a DSM-IV-TR diagnosis should be given a DSM-5 diagnosis of ASD.

Next, members of the author group who have expertise in epidemiology and developmental psychology conducted a literature review and utilized clinical judgment to determine the probability of a child meeting DSM-5 surveillance status based on DSM-IV-TR surveillance status and/or the presence of a documented ASD diagnosis in evaluation records. For instance, some studies found that most children diagnosed with DSM-IV-TR autistic disorder met criteria for DSM-5 ASD [15,16]; we therefore estimated the probability that a child with a documented diagnosis of autistic disorder would meet DSM-5 surveillance status as “high” and sampled 10% of those records. In contrast, there is variability in whether children with DSM-IV-TR Asperger disorder or PDD-NOS met criteria for DSM-5 ASD; we therefore estimated the probability that a child with a documented diagnosis of ASD would meet DSM-5 surveillance status as “uncertain” and sampled 100% of those records [18–20]. In sum, a priori, descriptive probabilities based on previous literature and clinical expertise were used to develop a stratified sampling approach to identify records for DSM-5 record-review coding (Table 2). Sampling was further stratified on race/ethnicity to ensure that the demographic distribution of DSM-5 samples was similar to the overall demographic distribution of children reviewed for ASD for the 2012 surveillance year using the DSM-IV-TR criteria.

Six clinicians established inter-rater reliability on the DSM-5 coding scheme. These six clinicians then coded records according to DSM-5 criteria based on the stratified sampling approach outlined in Table 2. Clinicians were blind to DSM-IV-TR surveillance status, and to the stratum from which each individual record was drawn. However, clinicians may have been aware of a previous ASD diagnosis if described within the abstracted text sent to the clinician for review. Clinicians met twice during the eight-week coding period to discuss inter-rater reliability for a common record reviewed by each clinician. Agreement on ASD surveillance status among clinicians was 100% for both of these reliability exercises.

### Analytic methods

All analyses were weighted to reflect the sampling strategy outlined in Table 2. Descriptive analyses were used to report the number of children who met the following surveillance status: (1) both DSM-IV-TR and DSM-5, 2) neither DSM-IV-TR nor DSM-5, (3) DSM-IV-TR, but not DSM-5, and (4) DSM-5, but not DSM-IV-TR. The kappa statistic assessed agreement between DSM-IV-TR surveillance status and the DSM-5 surveillance status of ASD.

Chi-square analyses compared the proportion of children who met only DSM-IV-TR surveillance status to those who met only DSM-5 surveillance status on the following variables: age at first evaluation abstracted (three years or younger or older than three years), autism classroom in public school (yes or no), developmental concern noted by three years of age (yes or no), documented ASD diagnosis (yes or no), ID (yes, no, or unknown), race/ethnicity (white non-Hispanic; non-white non-Hispanic; Hispanic), and sex (boy or girl). Omnibus chi-square values are reported.

## Results

Clinicians reviewed records of 1,542 children for MADDSP surveillance year 2012 according to DSM-IV-TR criteria. Of these, 77.4% were male and 37.0% were Non-Hispanic White, 37.9% were Non-Hispanic Black, 11.8% other or missing race, and 13.3% were Hispanic. Records of 536 children were chosen for DSM-IV-TR versus DSM-5 comparison based on the sampling scheme previously described (Table 2). Table 3 shows that, when sample weights were applied, 46.0% children met both DSM-IV-TR and DSM-5 surveillance status, 44.0% met neither the DSM-IV-TR nor DSM-5 surveillance status, 4.0% met DSM-IV-TR status, but not DSM-5 status, and 6.0% met DSM-5 status, but not DSM-IV-TR status of ASD. This represents 90.0% concordance and 10.0% discordance on DSM-IV-TR/DSM-5 surveillance of ASD. Kappa agreement was 0.80 (p < .01), which indicates excellent agreement. Kappa agreement was reduced to 0.63 (p < .01), which indicates good agreement, when DSM-5 cases with only a previous ASD diagnosis were excluded from surveillance counts.

**Table 3:** Number and percent of children who met autism spectrum disorder (ASD) surveillance status based on criteria outlined in the Diagnostic and Statistical Manual of Mental Disorders-Fourth Edition-Text Revision (DSM-IV-TR) and Fifth Edition (DSM-5) when sample weights were applied.

	DSM-IV-TR ASD	DSM-IV-TR NonASD	
DSM-5 ASD	709 (46.0%)^a^	91 (6.0%)^b^	800
DSM-5 NonASD	62 (4.0%)	680 (44.0%)	742
	771	771	1,542

^a^Of these, 88 (12.4%) met DSM-5 criteria solely based on a previous ASD diagnosis

^b^Of these, 22 (24.2%) met DSM-5 criteria solely based on a previous ASD diagnosis

Children who met only DSM-IV-TR surveillance status were compared to children who met only DSM-5 surveillance status on a number of child characteristics (Table 4). A higher proportion of children who met only DSM-IV-TR surveillance status, compared with those who met only DSM-5 surveillance status, had a developmental concern noted by three years of age and a developmental evaluation conducted by three years of age. By contrast, a higher proportion of children who met only DSM-5 surveillance status, compared with those who met only DSM-IV-TR surveillance status, were eligible for autism services at a public school or had an ASD diagnosis documented in service records. There were no differences in group proportions based on child race/ethnicity, child sex, or presence of ID.

**Table 4:** Characteristics of children who met autism spectrum disorder (ASD) surveillance status based only on the Diagnostic and Statistical Manual of Mental Disorders-Fourth Edition-Text Revision (DSM-IV-TR) criteria versus only on Fifth Edition (DSM-5) criteria when sample weights were applied.

Characteristic	Met Only DSM-IV-TR Surveillance Status N = 62 Weighted %	Met Only DSM-5 Surveillance Status N = 91 Weighted %	P value for χ^2^
Age at first health or education evaluation identified			< .01
Older than three years	67.7	97.8	
Three years or younger	32.3	2.2	
Autism classroom in public school			.03
No	79.0	63.7	
Yes	21.0	36.3	
Delays before three years of age			< .01
No	19.4	61.5	
Yes	80.6	38.5	
Documented ASD diagnosis			.01
No	80.6	62.6	
Yes	19.4	37.4	
Intellectual disability (IQ ≤ 70)			.94
No	48.4	47.8	
Yes	25.8	16.7	
Unknown	25.8	35.6	
Race/ethnicity			.66
White	37.1	33.7	
Non-white	41.9	49.4	
Hispanic	21.0	16.9	
Sex			.42
Boy	83.9	81.3	
Girl	16.1	18.7	

## Discussion

Similar to ADDM data reported for the 2014 surveillance period, we found substantial agreement between DSM-IV-TR and DSM-5 surveillance status of ASD in a sample of records reviewed for the 2012 surveillance period. Children who met only DSM-IV-TR surveillance status and those who met only DSM-5 surveillance status did not differ in terms of child race/ethnicity, child sex, or presence of ID. These results suggest that the surveillance status of ASD based on DSM-5 criteria is largely comparable to that based on DSM-IV-TR criteria, and identifies children with similar demographic and intellectual characteristics. One implication of these findings is that future reports of DSM-5 prevalence estimates may be considered within the context of historically presented DSM-IV-TR prevalence estimates given the level of agreement and similar characteristics among children identified by both surveillance methods.

Of course, agreement between DSM-IV-TR versus DSM-5 surveillance status is dependent on the case definition(s) employed by the surveillance system. CDC incorporated a previous ASD diagnosis into the DSM-5 case definition to adhere to new guidance offered in the diagnostic manual and reflect community practice. However, between 12.4% and 24.2% of those who met DSM-5 surveillance status did so based solely on a previous ASD diagnosis, and agreement between DSM-IV-TR and DSM-5 was reduced when these children were excluded from the DSM-5 surveillance definition. This finding could have implication for future surveillance efforts. On the one hand, fewer children could meet DSM-5 surveillance status in future years because they no longer qualify for a DSM-IV-TR diagnosis of ASD. Conversely, healthcare providers may begin to document more DSM-5 behavioral symptoms in service records to justify an ASD diagnosis after changes in diagnostic criteria. This latter consideration is especially important since records reviewed for this study were from 2012 –one year before DSM-5 was published. The ADDM Network is uniquely equipped to monitor these potential outcomes over time, and evaluate how a previous ASD diagnosis and service delivery influences DSM-5 surveillance status.

Our results differ from recent clinical analyses that indicate fewer children meet DSM-5 criteria for ASD than DSM-IV-TR criteria for ASD in similar service settings [18,22]. However, differences in clinical and surveillance definitions may help explain the discrepancy in findings. The ADDM surveillance definition of ASD is based on all information contained in health and education records from the first evaluation identified until the child is 8 years of age. In contrast, current clinical diagnoses may represent child functioning at one point in time and often in one service setting. It is therefore prudent for clinicians to consider developmental history and previous diagnoses when evaluating children for a DSM-5 diagnosis of ASD. Future research is needed to explore how current versus historical functioning influence clinical impressions and surveillance results.

There were few (i.e., 10.0%) of children in our sample with a discordant DSM-IV-TR and DSM-5 surveillance status of ASD. Therefore, comparisons of children who met only one of the ASD surveillance definitions represent a minority of the sample. Nonetheless, children who met only DSM-IV-TR surveillance status (4.0%) were more likely to have developmental concerns and evaluations in the first three years than those who met only DSM-5 surveillance status. These results indicate DSM-IV-TR criteria may detect few children with long-standing social-communication concerns that do not meet the number and pattern of deficits specified in DSM-5. Again, taking into account developmental history in addition to current functioning may help alleviate some concerns regarding a reduced number of ASD diagnoses under DSM-5 when compared to DSM-IV-TR.

Children who met only DSM-5 surveillance status (6.0%) were more likely to have been receiving autism-related services or previously diagnosed with ASD than children who met only DSM-IV-TR surveillance status. This result is not surprising given that DSM-5 diagnostic criteria includes a previous DSM-IV-TR diagnosis of autistic disorder, Asperger disorder, or PDD-NOS.

It may seem counterintuitive that children could meet DSM-5 but not DSM-IV-TR surveillance status if DSM-5 criteria are by apparent definition more stringent than DSM-IV-TR criteria. Detailed review of a few examples suggest that this result may be a consequence of the amount and type of information contained in surveillance records coupled with differences between DSM-IV-TR and DSM-5 conceptualizations of ASD. For instance, one child had almost all of DSM-IV-TR and DSM-5 criteria noted in surveillance records but did not have a developmental delay noted before three years of age or an autism discriminator (i.e., a behavior that distinguishes children with ASD from children with other developmental delays). A developmental delay before three years of age is required for autism classification under DSM-IV-TR but is not required for ASD classification under DSM-5. Specifically, DSM-5 states that “symptoms must be present in the early developmental period but may not become fully manifest until social demands exceed limited capacities, or may be masked by learned strategies in later life” [1]. An autism discriminator is required to meet ASD surveillance status–for those who do not meet the criteria for autism–under DSM-IV-TR but not DSM-5. Another example is a child who had three social deficits noted in service records, along with repetitive behaviors and sensory deficits. This child met DSM-5 surveillance status but not DSM-IV-TR surveillance status of autism or ASD because there were not at least six criteria or an autism discriminator, respectively, recorded (and sensory deficits are not considered in DSM-IV-TR diagnostic criteria).

There are limitations associated with record-review surveillance and these particular analyses. First, record-review surveillance relies on information contained in surveillance records and does not include an in-person evaluation of the child. Second, this was one of the first applications of the ADDM coding scheme developed for DSM-5 surveillance and was considered a pilot project for future DSM-5 surveillance efforts. Third, we did not have enough resources to code the entire MADDSP 2012 sample according to both DSM-IV-TR and DSM-5 criteria and, therefore, relied on a sampling framework that could introduce variability in estimates. Finally, records were from the 2012 surveillance period which was one year before the DSM-5 was published.

These limitations do not supersede the benefits of our analyses. This is one of the first studies to report a high level of agreement between surveillance status based on DSM-IV-TR and DSM-5 criteria for ASD. Moreover, our findings suggest that these two surveillance methods identify children with similar demographic and intellectual characteristics. Few children in our sample had a discordant surveillance status based on DSM-IV-TR and DSM-5 criteria. Those with a discrepant surveillance status could be due to current versus historical presentation and the nature of the behaviors described in service records. ADDM record-review surveillance is uniquely equipped to continue to evaluate the influence of diagnostic criteria–and a previous ASD diagnosis–on ASD surveillance status and prevalence estimates in large and diverse samples of children. This study is therefore an important first step to future ASD surveillance projects and how DSM-5 prevalence estimates can be considered within the context of historically presented DSM-IV-TR prevalence estimates.

## References

[1] American Psychiatric Association. Diagnostic and statistical manual of mental disorders 5th ed Arlington VA: American Psychiatric Publishing; 2013.

[2] Autism and Developmental Disabilities Monitoring Network Surveillance Year 2002 Principal Investigators; Centers for Disease Control and Prevention. Prevalence of autism spectrum disorders: Autism and Developmental Disabilities Monitoring Network, six sites, United States, 2000. MMWR Surveill Summ. 2007;56(1):1–11. 17287714

[3] Baio J, Wiggins LD, Christensen DL, Maenner M, Daniels J, Warren Z, et al Prevalence of Autism Spectrum Disorder Among Children Aged 8 Years—Autism and Developmental Disabilities Monitoring Network, 11 Sites, United States, 2014. MMWR Surveill Summ. 2018;67(6):1–23. doi: 10.15585/mmwr.ss6706a1 29701730 10.15585/mmwr.ss6706a1 PMC5919599

[4] Rice CE, Baio J, Van Naarden Braun K, Doernberg N, Meaney FJ, Kirby RS; ADDM Network. A public health collaboration for the surveillance of autism spectrum disorders. Paediatr Perinat Epidemiol. 2007;21(2):179–190. 10.1111/j.1365-3016.2007.00801.x 17302648 10.1111/j.1365-3016.2007.00801.x

[5] American Psychiatric Association. Diagnostic and Statistical Manual of Mental Disorders 4th ed, text revision. Washington, DC: American Psychiatric Association; 2000.

[6] Nonkin-Avchen R, Wiggins LD, Devine O, Van Naarden Braun K, Rice CE, Hobson NC, Schendel D, Yeargin-Allsopp M. Evaluation of a record-review surveillance system used to determine the prevalence of autism spectrum disorder. J Autism Dev Disord. 2011;41:227–236. 10.1007/s10803-010-1050-7 20568003 10.1007/s10803-010-1050-7

[7] Van Naarden Braun K, Pettygrove S, Daniels J, et al; Centers for Disease Control and Prevention. Evaluation of a methodology for a collaborative multiple source surveillance network for autism spectrum disorders: Autism and Developmental Disabilities Monitoring Network, 14 sites, United States, 2002. MMWR Surveill Summ. 2007;56(1):29–40. 17287716

[8] Amir RE, Van den Veyver IB, Wan M, et al Rett syndrome is caused by mutations in X-linked MECP2, encoding methyl-CpG-binding protein 2. Nat Genet. 1999;23:185–188. 10.1038/13810 10508514 10.1038/13810

[9] Autism and Developmental Disabilities Monitoring Network Surveillance Year 2002 Principal Investigators; Centers for Disease Control and Prevention. Prevalence of autism spectrum disorders: autism and developmental disabilities monitoring network, 14 sites, United States, 2002. MMWR Surveill Summ. 2007;56(1):12–28. 17287715

[10] Centers for Disease Control and Prevention.Brief update: prevalence of autism spectrum disorders (ASDs): Autism and Developmental Disabilities Monitoring (ADDM) Network, United States, 2004. MMWR Surveill Summ. 2009;58(10):21–24. 20023608

[11] Autism and Developmental Disabilities Monitoring Network Surveillance Year 2006 Principal Investigators, Centers for Disease Control and Prevention. Prevalence of autism spectrum disorders: Autism and Developmental Disabilities Monitoring Network, United States, 2006 [published correction appears in MMWR Surveill Summ. 2010;59(30):956]. MMWR Surveill Summ. 2009;58(10):1–20. 20023608

[12] Autism and Developmental Disabilities Monitoring Network Surveillance Year 2008 Principal Investigators, Centers for Disease Control and Prevention. Prevalence of autism spectrum disorders: Autism and Developmental Disabilities Monitoring Network, 14 sites, United States, 2008. MMWR Surveill Summ. 2012;61(3):1–19. 22456193

[13] Autism and Developmental Disabilities Monitoring Network Surveillance Year 2010 Principal Investigators, Centers for Disease Control and Prevention. Prevalence of autism spectrum disorders among children 8-years: Autism and Developmental Disabilities Monitoring Network, 11 sites, United States, 2010. MMWR Surveill Summ. 2014;63(SS02):1–21. 24670961

[14] Christensen DL, Baio J, Van Naarden Braun K, Bilder D, Charles J, Constantino JN, Daniels J, Durkin MS, Fitzgerald RT, Kurzius-Spencer M, Lee L-C, Pettygrove S, Robinson C, Schulz E, Wells C, Wingate MS, Zahorodny W, Yeargin-Allsopp M. Prevalence and Characteristics of Autism Spectrum Disorder Among Children Aged 8 Years—Autism and Developmental Disabilities Monitoring Network, 11 Sites, United States, 2012. MMWR Surveill Summ. 2016;65(3):1–23. doi: 10.15585/mmwr.ss6503a1 27031587 10.15585/mmwr.ss6503a1 PMC7909709

[15] Huerta M, Bishop S, Duncan A, Hus V, Lord C. Application of DSM-5 criteria for autism spectrum disorder to three samples of children with DSM-IV diagnoses of pervasive developmental disorder. Am J Psych. 2012: 169: 1056–1064. 10.1176/appi.ajp.2012.12020276 PMC6003412 23032385

[16] Kim YS, Fombonne E, Koh YJ, Kim SJ, Cheon KA, Leventhal BL. A comparison of DSM-IV pervasive developmental disorder and DSM-5 autism spectrum disorder in an epidemiologic sample. J Am Acad Child Adolesc Psychiatry. 2014: 53(5): 500–508. 10.1016/j.jaac.2013.12.021 24745950 10.1016/j.jaac.2013.12.021 PMC4058782

[17] Maenner MJ, Rice CE, Arneson CL, Cunniff C, Schieve LA, Carpenter LA, Van Naarden Braun K, Kirby RS, Bakian AV, Durkin MS. Potential impact of DSM-5 criteria on autism spectrum disorder prevalence estimates. JAMA Psych. 2014;71(3):292–300. 10.1001/jamapsychiatry.2013.3893 PMC4041577 24452504

[18] Matson JL, Kozlowski AM, Hattier MA, Horovitz M, Sipes M. DSM-IV versus DSM-5 diagnostic criteria for toddlers with autism. Dev Neuro. 2012: 15(3): 185–190. 10.3109/17518423.2012.672341 22582849

[19] Mattila ML, Kielinen M, Linna SL, et al Autism spectrum disorders according to DSM-IV-TR and comparison with DSM-5 draft criteria: an epidemiological study. J Am Acad Child Adolesc Psychiatry. 2011: 50(6): 592–583. 10.1016/j.jaac.2011.04.001 21621142

[20] McPartland JC, Reichow B, Volkmar FR. Sensitivity and specificity of proposed DSM-5 diagnostic criteria for autism spectrum disorder. J Am Acad Child Adolesc Psychiatry. 2012: 51(4): 383–368. 10.1016/j.jaac.2012.01.007 PMC3424065 22449643

[21] Tsai LY. Impact of DSM-5 on epidemiology of autism spectrum disorder. Res Autism Spectr Disord. 2014;8(11):1454–1470.

[22] Hartley-McAndrew M, Mertz J, Hoffman M, Crawford D. Rates of autism spectrum disorder diagnosis under the DSM-5 criteria compared to DSM-IV-TR criteria in a hospital-based clinic. Ped Neurology. 2016;57: 34–38. 10.1016/j.pediatrneurol.2016.01.012 26869267

